# Factors that influence adherence to aspirin therapy in the prevention of preeclampsia amongst high-risk pregnant women: A mixed method analysis

**DOI:** 10.1371/journal.pone.0229622

**Published:** 2020-02-27

**Authors:** Renuka Shanmugalingam, Zelalem Mengesha, Stephanie Notaras, Pranee Liamputtong, Ian Fulcher, Gaksoo Lee, Roshika Kumar, Annemarie Hennessy, Angela Makris

**Affiliations:** 1 Department of Renal Medicine, South Western Sydney Local Health District, Liverpool, NSW, Australia; 2 School of Medicine, Western Sydney University, Penrith, NSW, Australia; 3 Vascular Immunology Group, Heart Research Institute, University of Sydney, Newtown, NSW, Australia; 4 Women’s Health Initiative Translational Unit (WHITU), Ingham Institute For Applied Medical Research and South Western Sydney Local Health District, Liverpool, NSW, Australia; 5 Research and Social Policy Team, Uniting Australia, Sydney, NSW, Australia; 6 School of Health Sciences and Translational Health Research Institute, Western Sydney University, Penrith, NSW, Australia; 7 Department of Obstetrics and Gynaecology, Liverpool Hospital, Liverpool, NSW, Australia; 8 School of Medicine, University of New South Wales, Sydney, NSW, Australia; ESIC Medical College & PGIMSR, INDIA

## Abstract

**Background:**

Non-adherence with medications in pregnancy is increasingly recognized and often results in a higher rate of preventable maternal and fetal morbidity and mortality. Non-adherence with prophylactic aspirin amongst high-risk pregnant women is associated with higher incidence of preeclampsia, preterm delivery and intrauterine growth restriction. Yet, the factors that influences adherence with aspirin in pregnancy, from the women’s perspective, remains poorly understood.

**Objective:**

The study is aimed at understanding the factors, from the women’s perspective, that influenced adherence with prophylactic aspirin in their pregnancy.

**Study design:**

A sequential-exploratory designed mixed methods quantitative (n = 122) and qualitative (n = 6) survey of women with recent high-risk pregnancy necessitating antenatal prophylactic aspirin was utilized. Women recruited underwent their antenatal care in one of three high-risk pregnancy clinics within the South Western Sydney Local Health District, Australia. The quantitative study was done through an electronic anonymous survey and the qualitative study was conducted through a face-to-face interview. Data obtained was analysed against women’s adherence with aspirin utilizing phi correlation (φ) with significance set at <0.05.

**Results:**

Two key themes, from the women’s perspective, that influenced their adherence with aspirin in pregnancy were identified; (1) pill burden and non-intention omission (2) communication and relationship with health care provider (HCP). Pill burden and its associated non-intentional omission, both strongly corelated with reduced adherence (Φ = 0.8, p = 0.02, Φ = 0.8, p<0.01) whilst the use of reminder strategies minimized accidental omission and improved adherence (Φ = 0.9, p<0.01). Consistent communication between HCPs and a good patient-HCP relationship was strongly associated with improved adherence (Φ = 0.7, p = 0.04, Φ = 0.9, p = <0.01) and more importantly was found to play an important role in alleviating factors that had potentials to negatively influence adherence with aspirin in pregnancy.

**Conclusion:**

This study identified factors that both positively and negatively influenced adherence with aspirin amongst high-risk pregnant women. Is highlights the importance in recognizing the impact of pill burden in pregnancy and the need to counsel women on the utility of reminder strategies to minimize non-intentional omission. Importantly, it emphasizes on the importance of a positive patient-HCP relationship through effective and consistent communication to achieve the desired maternal and fetal outcomes.

## Introduction

Medication adherence in a health care setting is defined as the extent to which an individual takes medications and executes lifestyle changes in accordance to the recommendations from a health care provider [1, 2]. Non-adherence with medications, either by delayed or omitted doses, is reported in up to 50% of non-pregnant patients with chronic illness [3, 4] and is associated with higher morbidity and mortality [2, 4]. Non-adherence to medications during pregnancy is reported to occur in 40–60% patients and is associated with both significant maternal and fetal co-morbidities [5–7]. Pregnant women view prescribed medications differently and adherence with medications has been found to vary with the state of disease in pregnancy, uncertainty of the benefit of the medication, expected side effects and fear of teratogenic effects of medications [8–10]. This issue is particularly prominent with the use of prophylactic and maintenance therapy in managing maternal comorbidities, especially when the acuity of the maternal medical issue is not present [5, 11, 12].

Prophylactic use of aspirin is recommended in women who are at risk of developing preeclampsia and has been shown to have a risk reduction of 60–80% [13–15]. However, recent data suggest that the prophylactic benefit is best observed with ≥90% adherence with aspirin [16]. The problem, however, lies in adherence with aspirin therapy in pregnancy [17–19]. Our observational cohort study, which assessed for adherence quantitatively through plasma salicyclic acid detection and platelet function analyser-100 (PFA-100) assessment, demonstrated that 44% of high-risk women were <90% adherent with aspirin therapy [20]. Women with inadequate adherence demonstrated aa higher rate of both early and late onset preeclampsia with a higher rate of intrauterine fetal growth restriction (IUGR) and preterm delivery in comparison to women who were ≥90% adherent with aspirin therapy [20, 21]. This demonstrated that non-adherence to prophylactic aspirin increases the risk of potentially avoidable maternal and fetal complications in pregnancy.

Multiple factors are known to contribute towards both intentional and non-intentional non-adherence with medications in pregnancy [9, 10, 22]. However, an assessment on influencers of non-adherence, particularly with the use of prophylactic aspirin in pregnancy, has not been undertaken. Our study was aimed at examining the factors, from a patient’s perspective, that influences adherence with prophylactic aspirin in pregnancy and identifying factors that may enhance adherence and thus improve pregnancy outcomes.

## Method

We utilized an sequential-exploratory designed mixed methods approach, combining both quantitative and qualitative analysis [23]. Ethics approval for this study was obtained from the Ethics and Research Committee of South Western Sydney Local Health District NSW, Australia (HE 16/184). Women provided written informed consent to participate.

High-risk pregnant women who were on aspirin as part of a multi-centre longitudinal cohort study [20] within the South Western Sydney Local Health District (SWSLHD), NSW, Australia, were invited to participate in an online survey (SurveyMonkey^®^). Women included in this study were risk stratified as high-risk for preeclampsia based on the current guidelines [24, 25] and were prescribed aspirin (100mgs or 150mgs) by their treating clinicians. Women who were of non-English speaking background and who were unable to provide written informed consent were excluded.

Electronic invitations, via email, were sent out to 154 women, from October 2018 –June 2019, to participate in this anonymous, non-compulsory survey that consisted mainly of multichoice responses with additional free text boxes for additional responses. As part of the quantitative questionnaire, women were required to specify the extend of adherence (self-reported) with prescribed aspirin during their recent pregnancy. Anonymous response was utilized to facilitate honest and non-biased responses. Questions for the quantitative study were generated based on the validated adherence barrier questionnaire (ABQ) [26] and factors that are known to influence adherence with medications in pregnancy (S1 Table) [27, 28].

Women were also invited to express their interest in participating in a one-to-one, face-to-face interview for the qualitative assessment. Purposeful selection of the interview participants was done based on the participant’s obstetric history and biochemically observed adherence to aspirin [20, 21] to gain maximum variation in the sample. The qualitative study was designed based on the quantitative data with the aim of further exploring the data obtain. Open ended questions for the qualitative interview were generated based on the current literature on medication adherence in pregnancy in addition to the data obtained from the quantitative survey (S2 Table). The semi-structured interviews were conducted by a single experienced co-investigator who was not involved with the clinical care of these women during their pregnancy. The interviews were conducted on an average of 12 months post-partum and were audio-recorded with short key notes taken during interviews to allow for member checking at the end of the interview. Participants were provided with a verbal summary of the interview and the opportunity to request amendments to the interpretations. The duration of the interviews ranged from 45 to 75 minutes. Interviews were ceased at the point of data saturation, which was set at the point at which no new data emerged from interviews.

Adherence for the purposes of the later thematic analysis was defined based on graded self-reported adherence with aspirin of ≥90% for the duration of the pregnancy as specified in the quantitative survey. A value of ≥90% was based on current data [16]. Audio recorded interviews were transcribed verbatim. No preconceived codes or categories were used. A six-stage sequential qualitative analysis was undertaken in analysing the transcription with the use of NVivo^®^ (v.12 QRS International Pty Ltd) [29]. Transcripts were re-coded by a second investigator to ensure agreement. Results of the quantitative and qualitative studies were combined through a process of triangulation that enabled the investigators to connect and interpret both data sets simultaneously through convergence and corroboration with the use of NVivo^®^ [30, 31]. Phi correlation(φ) of data was done through SPSS (v.25 Chicago, IL, USA). Data was analysed using the appropriate test based on the data distribution and statistical significance was set at 0.05. A φ value of 0.7–1 is representative of a strong correlation, 0.3–0.69 of a moderate correlation and values of <0.3 are representative of a weak correlation.

## Results

Of the 154 invitations sent out, 122 women (79%) completed the survey. Thirty-six women expressed interest in participating in the qualitative study, of whom, 18 were identified through purposeful selection to attend the interviews that were conducted at Liverpool Hospital, NSW, Australia. Interviews were ceased at point of qualitative data saturation which was achieved after the sixth interview. Three of these 6 women in the qualitative study were noted to have >90% adherence in the longitudinal study [20] with <90% adherence in the remaining three women. Characteristics of the women invited to participate in the quantitative study and those in the qualitative study are described in Tables 1 and 2 respectively. The quantitative survey demonstrated that 65 (53%) of the women reported ≥90% adherence with aspirin in their pregnancy.

**Table 1:** Characteristics of participants invited to participate in quantitative study (*).

Characteristics	Quantitative study invitations (n = 154)
Age (years) **	33 (± 5.6)
Duration in months since delivery (months)**	12.4 (± 7.4)
Primigravity	28 (17%)
Secondary education level	19 (12%)
Tertiary education level (Higher and Vocational)	135 (88%)
Smoking in pregnancy	8 (5%)
Ethnicity	
Caucasian	78 (51%)
Middle Eastern	24 (16%)
South Asian	22 (14%)
South East Asian	19 (12%)
African	6 (4%)
Polynesian	3 (2%)
Australian Aboriginal	2 (1%)
Indication for aspirin in most recent pregnancy^§^	
Chronic hypertension	90 (53%)
Previous pre-eclampsia	74 (44%)
Renal disease	35 (21%)
Type 1 or Type 2 Diabetes	13 (8%)
Based on first trimester screening only	8 (5%)
Systemic lupus erythematoses	5 (3%)
Anonymously self-reported adherence of ≥90% with aspirin	65 (53%)^#^

*Given the anonymous nature of this survey the characteristics of the women who participated (79% of total women invited) could not be isolated. Presented here are the characteristics of all women invited to participate except for #, which is based on the response from women who participated in the survey.

**Mean with standard deviation (± SD). §Some women had more than one medical condition.

**Table 2:** Characteristics of participants in qualitative study.

Characteristics	Qualitative study participants (n = 6)
Age (years)*	30.3 (±3.7)
Duration since delivery (months)*	14.8 (±2.5)
Primigravity	1 (17%)
Secondary education level	2 (33%)
Tertiary education level (Higher and Vocational)	4 (67%)
Smoking in pregnancy	0 (0%)
Ethnicity	
Caucasian	4 (67%)
South Asian	1 (17%)
Middle Eastern	1 (17%)
Indication for aspirin in pregnancy**	
Chronic hypertension	2 (33%)
Type 1 or Type 2 Diabetes	1 (17%)
Renal disease	1 (17%)
Previous pre-eclampsia	4 (67%)

*Mean with standard deviation (±SD).

** Some women had more than one medical condition.

Based on the combined analysis, we identified factors that influenced women’s adherence with aspirin therapy in pregnancy. Results are present thematically: (1) Pill burden and non-intentional omission (2) Communication and relationship with health care providers (HCPs) (Table 3)

**Table 3:** Summary of factors that influenced adherence with prophylactic aspirin.

Themes	Factors	Effect on adherence with aspirin	P value*
**Pill burden and non-intentional omission**	Pill burden	Reduced	Φ = 0.8, p = 0.02
	Non-intentional omission	Reduced	Φ = 0.8, p<0.01
	Strategizes to minimize non-intentional omission	Improved	Φ = 0.9, p<0.01
**Communication and relationship with Health Care Providers (HCPs)**	Consistent messaging between HCPs	Improved	Φ = 0.7, p = 0.04
	Repeated re-enforcement by HCPs	Improved	Φ = 0.8, p = 0.02
	Influence of social media and women support group	Improved	Φ = 0.7, p = 0.03
	Positive relationship with HCPs	Improved	Φ = 0.9, p<0.01

*Based on phi coefficient relationship (φ)

### Pill burden and non-intentional omission: *“I had so many pills and aspirin was another pill*..*it was a struggle”*

On average, women in our study were on 3.5 (range 2–6) medications at any one time in their pregnancy with repeated dosing over 24 hours (average: 12 hourly). Of the women who missed more than 1 dose of aspirin (<90% adherent) in pregnancy, 42 (74%) reported pill burden as an issue (φ = 0.8, p = 0.02) and this was associated with a higher rate of patient reported non-intentional omission of aspirin (φ = 0.8, p<0.01). This was further evident in the qualitative data where women who reported non-intentional omission were on an average of 6 pills a day in comparison to women with good adherence with aspirin (average of 2 pills a day):

“*I was taking lots of other vitamins and things*, *so yeah*, *I don’t think I fully grasped how important it was to take aspirin every day*. *It was definitely not something I prioritized*. *I didn’t like taking medications in general*. *I feel like there was a lot towards the end*. *I got a little bit hysterical*.*” (Interview participant 1*, *aspirin non-adherent group*, *29 yo)*

“*To be honest with you*, *I was taking a lot*. *There was a time where I was taking eight per day; that included things like Elevit*, *Vitamin D*, *my blood pressure medication and aspirin*, *yeah*, *so a lot*. *I had to take my diabetic medication*, *Aspirin*, *Macrolide*, *Folate and Vitamin D and Calcium so yeah it was hard to keep track of all of it*.*” (Interview participant 4*, *aspirin non-adherent group*, *32 yo)*

Non-intentional omission was also strongly related to difficulty with taking aspirin at night as instructed by their doctor in 20 (35%) of women (φ = 0.8, p<0.01). The qualitative study further indicated that the need to adhere to specified timing of aspirin ingestion (bedtime dosing), along with pill burden was associated with increased non-intentional omission of aspirin:

“*I had all my medications besides my diabetes medication and aspirin cause I had to take that at specific times especially at night*. *When I got into bed and forgot to take all my medications*, *I went ‘I’m not getting back out of bed*. *I’m exhausted*. *Yeah I’m in bed for the night now*. *I know later on I’m going to get out of bed a million times*. *No I’m not getting out just to take medication’*.*” (Interview participant 4*, *aspirin non-adherent group*, *32 yo)*

A total of 65 (53%) women reported good adherence with aspirin either with no omission 45(37%) or only 1 omitted dose 20(16%) through the duration of their pregnancy. The strategies utilized to minimize accidental omission included the use of a mobile phone reminder application in 42(65%), pill box in 19(29%) and a medication routine 9(14%). The use of these strategies strongly correlated with aspirin adherence (φ = 0.9, p<0.01) and was supported by the qualitative responses in which women elaborated on the reminder strategies they utilized to overcome issues in relation to bedtime dosing of aspirin:

“*I had a reminder strategy*. *So usually I would take it when I brush my teeth*, *as a reminder*. *So I just made sure that they were there and when I went to brush my teeth*, *I would see it and take it*.*” (Interview participant 3*, *aspirin adherent group*, *34 yo)*

“*I put all my medications in my room in my drawer so I knew when I went to bed I pulled out the drawer and get all my medications out ready and yeah go to bed*. *I knew where all my medication was*, *and I had to take it*.*” (Interview participant 6*, *aspirin adherent group*, *30 yo)*

Therefore, whilst pill burden and non-intentional omission of medications were key factors that negatively influenced adherence, the use of reminder strategies was an effective influencer in overcoming this.

### Communication and relationship with Health Care Providers (HCPs): *“I had a good rapport with my doctor early on which helped me trust the healthcare system”*

Consistent communication between the women’s HCP positively impacted their uptake of aspirin. Most women, 101(83%), were first advised to take aspirin by their renal/obstetric physician, 33 (27%) by an obstetrician or midwives and 20 (16%) by general practitioners or based on previous recommendation. Of these women, 59 (48%) discussed the use of aspirin with a second HCP. Consistent message from HCPs on the indication of aspirin in pregnancy strongly correlated to women’s uptake and adherence with aspirin (φ = 0.7, p = 0.04). The importance of consistent messaging and the negative impact of inconsistent messaging between HCPs was evident through the qualitative data in which women elaborated on how to information they obtained from multiple HCPs influenced their adherence with aspirin (both positively and negatively):

“*When I was told by the first doctor*, *I was still a bit sceptical and it’s only when I saw the second and third doctor*, *it sunk in and I thought*, *it must be important as they are all saying the same thing*. *It then made sense*. *It works well when doctors communicate the same thing*, *it gives us confidence*.*” (Interview participant 2*, *aspirin adherent group*, *34 yo)*

“*The chemist told me that I should not take aspirin while I was pregnant despite my doctor's advice*. *This made my husband very concerned and discouraged me from taking the aspirin (Interview participant 1*, *aspirin non-adherent group*, *29 yo)*

Additionally, 52(43%) of women reported that repeated re-enforcement on adherence with aspirin by HCPs emphasized on its importance. This strongly correlated with adherence (φ = 0.8, p = 0.02) and was supported by the qualitative data:

“*I remember my doctor saying not to forget to take my medications*, *especially the aspirin*, *so actually I do recall her saying that to me and made it think it must be important for her to say that*.*” (Interview participant 6*, *aspirin adherent group*, *30 yo)*

Communication on the use of aspirin in pregnancy with other high-risk women via social media and forums was found to strongly correlate with uptake and adherence with aspirin in 33(27%) of women (φ = 0.7, p = 0.03). This was also evident in the qualitative data in which these women elaborated on the positive impact of social media in reassuring them on the use of aspirin in pregnancy:

“*Speaking to other women that have been through it (preeclampsia) and that are going through it–you know finding friends who are on or who have taken aspirin in pregnancy*, *who are going through similar things gave me comfort in taking it*.*” (Interview participant 6*, *aspirin adherent group*, *30 yo)*

Women’s good relationship with their HCPs played an important role in alleviating factors that had potentials to negatively influence adherence with aspirin in pregnancy. These factors included inconsistent messaging among HCP, especially from pharmacist which was reported in 36 (30%) of women, concerns regarding maternal and fetal side effects of aspirin 39 (32%) and discouragement from family and friends 18 (15%). However, none of these factors significantly correlated with adherence (φ = 0.1–0.4) as 111 (91%) of women reported having a good relationship with their primary HCPs and were happy to discuss their concerns on the use of aspirin in pregnancy (φ = 0.9, p<0.01). This was further corroborated by women who participated in the interviews:

“*The chemist kept telling me that I should not take aspirin while I was pregnant despite my doctor's advice*. *This made my husband and mother very concerned and they discouraged me from taking the aspirin*. *My husband was unhappy and came with me to my appointment to talk about this with my doctor*. *My doctor spent a lot of time to talk to us about it and put our mind at ease*. *She also called the chemist after we left*.*” (Interview participant 6*, *aspirin adherent group*,*30 yo)*

“*My previous pregnancy was horrible because I never had that connection with my doctor*. *So you know*, *there was a difference this time*, *the relationship that I had with my doctor made a huge difference*. *They had time to listen and answer my question*. *I knew I was under better care*. *I had trust in them*.*” (Interview participant 3*, *aspirin adherent group*, *34)*

## Discussion

Our study identified two key themes, from a patient’s perspective, that influenced adherence with prophylactic aspirin in pregnancy: 1) Pill burden and non-intentional omission and 2) Communication and positive relationship with HCPs (Table 3). We previously demonstrated that, from the women’s demographic characteristic perspective, women who had previous preeclampsia and tertiary level education had a higher rate of adherence with aspirin [20, 21].

Pill burden and non-intentional omission of medications are commonly associated with non-adherence in both pregnant and non-pregnant patients [2, 5, 25]. Our study demonstrated that these two factors were strongly correlated with suboptimal aspirin adherence, particularly when women were put on a bedtime regimen. In keeping with this, other studies have described restrictions around dosing requirements such as ‘take with food’, ‘take with an empty stomach’, ‘take away from other medications’ and ‘take at night’ as common contributing factors towards non-intentional medication omission [32, 33]. However, the use of electronic reminders and pill boxes as reminders has been shown to minimize accidental medication omission [34] and improve adherence [22]. Our study, similarly, demonstrated a strong positive correlation between the use of reminder strategies and adherence with aspirin therapy that can be used to overcome non-intentional omission with bedtime aspirin, particular given the growing recommendation for bedtime aspirin [13, 32].

Importantly, our study reflects the importance of communication and a positive relationship between the woman and her HCPs in achieving the desired level of aspirin adherence and clinical outcomes. Pregnant women view the use of medications in pregnancy differently compared to when they are not pregnant. They often question the need for medication in pregnancy, mainly due to concerns over potential teratogenic effects [8, 9]. Therefore, unsurprisingly, a woman’s understanding of the need for her medications influences her attitude towards the adherence in pregnancy and this is directly influenced by the way the need for the medication was communicated by her HCP [8]. Specific to this study, the importance of effective communication between HCPs and patients in discussing the need for aspirin and to discuss concerns regarding its use, was evident. The current literature supports the importance of a strong therapeutic relationship and trust in HCPs and patient’s behaviour towards therapy [33, 34]. This relationship is often found to be closely related to the clinician’s interpersonal skill, ability to provide clear explanation of correlation between therapy and disease, ability to inspire patient’s trust and adequately address patient’s concerns [35–38]. In our study, the relationship and interaction between the women and their HCP not only helped them understand the need for aspirin but also allowed for discussions on their concerns with regards to the use of aspirin and provide them with assurance, allowing for an improvement with adherence with prophylactic aspirin.

Similarly, re‐enforcing the importance of medications and checking for adherence with therapy have been also shown to improve patient’s adherence [37] and in keeping with the literature, women in our study resonated this association. Additionally, the use of social media as a domain of support network with other women has been shown to provide reassurance and help improve women’s understanding on the need for therapy in pregnancy [39, 40]. This has been shown to improve adherence with the recommended therapy in pregnancy [39]. In keeping with this, our study demonstrated a positive influence of social media and support group in facilitating adherence with aspirin in pregnancy.

To our knowledge, a mixed method study in analysing factors that influences adherence with aspirin in pregnancy, from a patient’s perspective has never been undertaken, hence, making this a novel study. Additionally, our patient population is vastly multicultural (Tables 1 and 2) with variation in education and socio-economic background. Our study, however, consist of a few limitations. Given the nature of both the quantitative and qualitative study, we excluded non-English speaking women given the inability to access a multilingual translational service for research. However, language barrier has been significantly demonstrated as a factor that influences adherence mainly due breakdown in communication [41]. This is often overcome with the use of a medical interpreter, however, the use of an interpreter may hamper forming a therapeutic relationship with HCPs and reduce the potential for patients to discuss their concerns [42]. Additionally, studies using electronic questionnaires carries a risk of selection bias towards the more literate population. Further mixed methodology analysis on the influence of medical literacy and language barrier, particularly in the migrant population will be instrumental. Our data may also be confounded by re-call bias given that the women participated with an average of 12.4 (± 7.4) months post-partum in the qualitative study and 14.8 (± 2.8 months) post-partum in the quantitative study. Additionally, whist it would have been ideal to unblind the participants in the quantitative study to use their data against their established biochemical evidence of adherence with aspirin from our longitudinal cohort study [20], we chose to allow for women to participate in the quantitative study anonymously to allow and encourage them to provide honest and unbiased response to better understand their experience on the use of aspirin in pregnancy. In doing so, we found that women’s self-reported adherence in this study matched what we observed biochemically in the longitudinal study. A non-anonymous patient-reported adherence assessment which we conducted as part of the longitudinal study however demonstrated that patient-reported adherence (in a non-anonymous form of qualitative assessment) only moderately correlated with their actual biochemical adherence, therefore indicating that patient-reported adherence is likely more accurate in an anonymous setting.

## Conclusion

Multiple clinical, psycho-social and health care related factors are known to influence adherence in pregnancy. Our study demonstrates factors within two key themes that influences the use of aspirin in the prevention of preeclampsia amongst high-risk pregnant women; (1) pill burden and accidental omission (2) communication and relationship with HCPs. It highlights the need for clinicians to be aware of the consequence of pill burden in pregnancy and importance of counselling patients on the utility of reminder strategies whilst applying repeated re-enforcement to minimize non-intentional omission of essential medications. Our study also demonstrates the crucial need for HCPs to recognize the importance of building a positive relationship with their patients through effective and consistent communication to achieve the desired maternal and fetal outcomes in pregnancy.

## Supporting information

S1 Table Questions used in quantitative study. (DOCX)

S2 Table Questions used in qualitative study. (DOCX)
