# Supplementary material for: Factors that influence adherence to aspirin therapy in the prevention of preeclampsia amongst high-risk pregnant women: A mixed method analysis
Source: PLoS One. 2020 Feb 27;15(2):e0229622. doi: 10.1371/journal.pone.0229622 (PMC7046289; doi:10.1371/journal.pone.0229622)
Supplement: S1 Table — (DOCX) [file pone.0229622.s001.docx]

**S1: Questions used in quantitative study**

| **Question** | **Question type** |
| --- | --- |
| How do you rate your understanding of why you were asked to take aspirin in your recent pregnancy? | Graded response |
| Who first advised you to take aspirin in your pregnancy? | Multi-choice response |
| How satisfied were you with your doctor’s/midwife’s explanation on the need for aspirin in your pregnancy? | Graded response |
| Can you please elaborate on what could have been done better with the explanation provided to you? | Free text response |
| Were you worried about taking aspirin in your pregnancy? | Yes/No response |
| What were your concerns with taking aspirin in pregnancy | Multi-choice response with free text for additional response |
| Did you feel comfortable discussing your concerns with your doctor/midwife? | Yes/No response |
| If no, why did you not feel comfortable discussing your concerns? | Free text response |
| How satisfied were you with the discussion with your pharmacist on the use of aspirin in pregnancy? | Graded response with additional free text response |
| How satisfied were you with your discussion with your obstetrician on the use of aspirin in pregnancy? | Graded response with additional free text response |
| Did you do your own research to understand the need for aspirin in pregnancy? | Yes/No response |
| What was your source of information | Multi-choice response |
| Did your research change your decision on taking aspirin in pregnancy | Yes/No with additional  free-text response |
| How often did you miss your aspirin in your pregnancy? | Graded response |
| What was the reason you missed your aspirin? | Multi-choice response with additional free text response |
| What strategies might have helped you take your aspirin consistently without missing it? | Multi-choice response with additional free text response. |
| Did you have to take other medications/vitamins in your recent pregnancy and if so, how many medications were you taking? | Multi-choice response with additional free text response |
