# Supplementary material for: Factors that influence adherence to aspirin therapy in the prevention of preeclampsia amongst high-risk pregnant women: A mixed method analysis
Source: PLoS One. 2020 Feb 27;15(2):e0229622. doi: 10.1371/journal.pone.0229622 (PMC7046289; doi:10.1371/journal.pone.0229622)
Supplement: S2 Table — (DOCX) [file pone.0229622.s002.docx]

**S2: Questions used in qualitative study**

| **Theme 1: Knowledge *(probing questions in italic)***  1) What is your understanding of preeclampsia    2) What is your understanding on why you were asked to take aspirin?  3) How did you feel about taking aspirin in your pregnancy?  *- were you worried? If so, what were your concerns?*  *- did you talk to anyone about your concern?*  *- what did your family and friends think about you being on aspirin?*  *- did you know anyone else on aspirin? Did this reassure you?*  4) Did you do your own research about using aspirin in pregnancy?  *- what was your source?*  *- was your source helpful? Did it scare you?*  *- what you have preferred as a source of information?*  *- what is your take on the information available on the internet?*  *- did your research change your decision on taking aspirin? if so, how and why?* |
| --- |
| **Theme 2: Compliance**  1) How many medications did you have to take during your pregnancy?  2) How did you feel about taking medications in pregnancy?  *- did you feel safe taking medications in pregnancy?*  *- did you feel like you knew enough about why you needed the medications?*  3) How often would you have missed medications  *- what was the reason you missed them?*  *- how often would you have missed aspirin?*  *- what was the reason for this?*  *- did you experience any side effects?*  *- we're there any restrictions that made it difficult? (Ie: taking it at a particular time/specific*  *instructions / tablet size or configuration)*    4) What would have helped you take your medications without missing them?  *- what strategies have you tried to use to minimize the times you missed your pills? Was this*  *effective? IF no, why?* |
| **Theme 3: Quality improvement**  1) Were you happy with the explanation your doctor provided with need for aspirin in your pregnancy?  *- did you discuss the use of aspirin in pregnancy with your renal doctors? Were you satisfied*  *with this interaction?*  *- did you discuss the use of aspirin with your obstetrician? We're you satisfied with this*  *interaction?*  *- did you discuss the use of aspirin with your GP? We're you satisfied with this interaction?*  *- did you discuss the use of aspirin with your pharmacist? We're you satisfied with this interaction?* |
